# Takotsubo cardiomyopathy in India and its electrocardiography (ECG) comparison to myocardial infarction

**DOI:** 10.1186/s43044-024-00453-x

**Published:** 2024-02-21

**Authors:** Tanisha Mishra, Rijushree Saha, Ganesh Paramasivam

**Affiliations:** Department of Cardiology, Manipal Academy of Higher Education, Kasturba Medical College, Manipal, Manipal, Karnataka 576104 India

**Keywords:** Takotsubo cardiomyopathy, Myocardial infarction, Electrocardiography, Stress cardiomyopathy

## Abstract

**Background:**

Owing to the limited research on Takotsubo Cardiomyopathy (TCM) in Asia, we aim to evaluate in detail the clinical profiles, lab parameters, investigations, and major adverse cardiovascular events (MACE) seen in patients with TCM in the Indian subcontinent. Additionally, we have compared the electrocardiographic findings of patients with TCM to those of patients with myocardial infarction (MI).

**Results:**

The average age of the patients affected was found to be 60 ± 11 years. Women (87.5%) and patients with hypertension (40%) were found to be at an increased risk of developing the syndrome. The most common presenting symptom was dyspnea (48%) following a trigger most commonly emotional (45%). ST elevation and significant T wave inversions were observed in 40% of patients with TCM. Echocardiography revealed a low left ventricular ejection fraction of 43 ± 9%. Coronary angiography was normal in 60%, the rest had mild/subcritical stenoses. The 6-month MACE was 20% and the mortality rate was 7.5%. Follow-up echocardiography of patients with TCM showed improvement in EF in 75% patients.

**Conclusions:**

TCM was majorly seen in postmenopausal women following an emotional trigger, but a variety of other triggers were noted. T-wave inversions in TCM follow a diffuse pattern in contrast to specific leads seen in MI. Normal or subcritical stenosis in coronaries at presentation, along with a low EF which improves on follow up provide greater evidence for the diagnosis of TCM.

## Background

Takotsubo Cardiomyopathy (TCM) is a clinical syndrome that causes temporary ballooning of the left ventricular apex along with elevated troponin levels, without significantly affecting coronary arteries [1, 2]. Initially discovered by a Japanese physician, the disease was named ‘Takotsubo Syndrome’ because of the similarity of apical ballooning to the shape of a Japanese octopus fishing pot [3]. Multiple emotional stressors ranging from the unexpected loss of a close relative, financial losses, receiving bad news (such as a recent cancer diagnosis), domestic violence, fierce arguments, etc., can precipitate TCM, thus leading to its other name, Broken Heart Syndrome [4]. It is commonly seen in postmenopausal women. TCM presents with shortness of breath, angina, ischemic electrocardiographic (ECG) changes, and regional wall abnormalities (RWMA) with elevated Troponin T and NT pro-BNP levels, which clinically mimics acute coronary syndromes (ACS) [5, 6]. The electrocardiographic changes, the systolic dysfunction, and the left ventricular ballooning seen in TCM are usually reversible with the trigger resolution [7].

Very little research has been conducted to compare TCM and Myocardial Infarction (MI) because TCM is a novel concept in India and Southeast Asia. Given that cardiovascular diseases rank as the leading cause of mortality in India [8], it is plausible that TCM might serve as an inconspicuous factor, owing to its tendency to be misdiagnosed as MI, thereby adding to the overall burden. This leads to mismanagement of TCM patients, with treatment traditionally reserved for MI, leading to adverse effects in patients, including progression to cerebral infarction and intracerebral hemorrhage [7, 9]. This necessitates the need for our study.

## Methods

### Study design, size and setting

This single-center, retrospective observational study included 40 patients diagnosed with TCM and 39 patients diagnosed with MI, in a tertiary care hospital in South India. The study was approved by the Institutional Ethics Committee (IEC) of the Author’s Institution. Clinical profiles, laboratory parameters, echocardiographic (ECHO), and coronary angiography (CAG) findings were analyzed for patients with TCM. A comparison of electrocardiographic (ECG) data of TCM patients was done with MI patients.

### Participants

The inclusion criteria were the same for TCM as well as MI. All patients aged 18–80 years, diagnosed with either TCM or MI, in the Department of Cardiology between January 2013 and January 2019 were included in the study.

### Data sources and measurement

Physical copies of the medical records of our study participants were obtained from the medical record department after obtaining appropriate permissions from the Medical Superintendent and the Head of the Department. From the records of patients with TCM, details about the clinical presentation, including clinical history, findings on physical examination, investigations including biochemical tests, ECG, ECHO, angiographic reports, and other medical and interventional treatments, were noted from the patient records. CAG images were obtained from the Department of Cardiology image database. In-hospital outcomes (Mortality, MACE) during index hospitalization were determined from hospital records. Post-discharge follow-up data for > 6 months were also obtained from the hospital records. The records of patients diagnosed with MI were similarly accessed and analyzed for comparison of ECG changes.

### Study objectives

1. To study the demographics, lab parameters, and investigations of patients diagnosed with Takotsubo Cardiomyopathy.
2. To compare ECG changes of Takotsubo Cardiomyopathy with those of Myocardial infarction
3. To study the rates of mortality and major adverse cardiovascular events (MACE) in-hospital and on follow-up (at 6 months) in patients with Takotsubo Cardiomyopathy.

### Variables

#### Diagnostic criteria

The diagnosis of TCM for the patients in our study was made using the Revised Mayo Clinic diagnostic criteria [10]

#### Outcome definitions

The study outcomes included major adverse cardiovascular events (MACE) and six-month mortality from all causes. MACE is defined as a composite of adverse events that predominantly considers all-cause death, nonfatal myocardial infarction, stroke, revascularization procedures, and hospitalization because of heart failure [11].

#### Follow up

Follow-up of the patients was done after six months, and data were obtained from the patient’s medical records.

### Statistical methods

The variables were categorized as continuous or non-continuous. For continuous variables, the mean and standard deviation were used. The variables that were not normally distributed have been represented as medians. Normality was calculated using the Shapiro–Wilk test. *P*-value was calculated to compare the ECG of patients with TCM and MI. A *p*-value of < 0.05 was taken as an indicator of statistical significance. To calculate the p-values, we used a two-sample t-test for continuous variables (such as ST elevation specific, ST elevation diffuse, ST depression specific, ST depression diffuse, T wave inversion specific, T wave inversion diffuse, poor R wave progression specific, poor R wave progression diffuse, and normal Q wave) and a Chi-square test for categorical variables (such as heart block, BBB, sinus tachycardia, and normal ECG). Statistical analysis was carried out using R software (version 4.3.1).

## Results

The 6-month mortality rate of the disease was calculated to be 7.5%, with cardiac arrest as the immediate cause of all. The differential diagnosis at the time of death was a cardiogenic shock, septicemia, and acute pulmonary edema. Around 20% of patients experienced major adverse cardiovascular events (MACE). The MACE was a composite of all-cause death (*n* = 4), stroke (*n* = 1), non-fatal myocardial infarction (*n* = 1) coronary revascularization procedures (*n* = 2) and hospitalization because of heart failure (*n* = 2). On a 6-month follow-up, the recurrence of TCM was seen in 2.7% of the total patients and the rate of repeat hospitalization was around 12.5% (Tables 1, 2 and 3).

**Table 1:** Clinical profiles and lab parameters

Clinical profile	Total (*n* = 40)
*Demographics*	
Age	60 ± 11
Women	35 (87.5%)
Men	5 (12.5%)
*Comorbid conditions*	
Diabetes	6 (15%)
Hypertension	16 (40%)
Peripheral vascular disease	1 (2.5%)
Others	7 (17.5%) [Including Bronchial Asthma 3 (7.5%); Chronic Obstructive Pulmonary Disease 2 (5%); Chronic Pancreatitis 1 (2.5); Lung Cancer 1 (2.5)]
No comorbid conditions	10 (25%)
*Clinical characteristics*	
Shortness of Breath	19 (48%)
Chest pain	11 (28%)
Others	10 (25%) [Including Weakness 4 (10%); Cough 2 (5%); Syncope 2 (5%); Fever 1 (2.5%); Abdominal Pain 1 (2.5%)]
*Triggers*	
Emotional trigger	18 (45%)
Infectious trigger	11 (27.5%)
Other triggers	11 (27.5%) [Including following pacemaker implantation for complete heart block 3 (7.5%); Malignancy 3 (7.5%); Ruptured Coronary Aneurysm 1 (2.5%); Post-Balloon Angiography 1 (2.5%); Bee Stings 1 (2.5%); Rheumatological Causes 1 (2.5%); Accidental Trauma 1 (2.5%)]
*Other factors*	
Time to Recovery (in days)	5 (3, 7)
Previous Hospitalization	10 (25%)
*Lab parameters*	
Troponin T (ng/mL) [Normal Range −0 to −0.04]	0.337 (0.174, 0.701)
NT Pro BNP (pg/mL) [Normal Range < 400]	2907 (752, 4899.5)

*BNP* brain natriuretic peptide

**Table 2:** Investigations

ECG parameters	Numerical value	ECG finding	*n* = 40
Heart Rate(bpm) [Normal range −60 to −100]	89 (71, 100)	Normal Sinus Rhythm	24 (60%)
		Bradycardia	10 (25%)
		Tachycardia	6 (15%)
QRS duration (ms) [Normal range −80 to −100]	100 (80, 100)	Prolonged QRS	14 (35%)
		Normal QRS	26(65%)
		QRS Axis deviation	6(62.5%)
QT Duration (ms) [Normal range −400 to −440]	390 (320, 490)		
QTc interval (ms) [Normal range −350 to −460]	481 (437, 530)	Normal QT	9(22.5%)
		Prolonged QT	15(37.5%)
RR interval(ms) [Normal range −600 to −1200]	650 (600, 825)		

ECHO Parameters (*n* = 40)	*n*%
LVEF [N = 55%—75%]	43 ± 9%
Abnormal Hypertrophy	5 (12.5%)
Right Ventricular Dysfunction	1 (2.5%)
Left Ventricular Dysfunction	18 (45%)
Valve Abnormalities (Stenosis or Regurgitation)	17 (42.5%)
Ischemic Heart Disease	8 (20%)

CAG parameters (*n* = 40)	*n* %
Mild stenosis	10 (25%)
Subcritical stenosis	6 (15%)
Normal CAG	24 (60%)

Follow up ECHO (*n* = 24)	*n* %
Resolution of wall motion abnormalities	18 (75%)
Improvement in LVEF	18 (75%)

*LVEF* left ventricular ejection fraction, *CAG* coronary angiography

**Table 3:** ECG characteristics and vitals comparison between patients affected by TCM and MI

ECG property (*x*)	Subtype	TCM (*n*1 total = 40)			MI (*n*2 total = 39)		***p***
		n_1_ total %	No of Leads in TCM	*n*_1_%	*n*_2_ total %	*n*_2_%	
ST elevation	Specific	16 (40%)	4 ± 2	13 (32.5%)	8 (20.51%)	3 (7.69%)	**0.043**
	Diffuse			3 (7.5%)		5 (12.82%)	0.474
ST depression	Specific	12 (30%)	4 ± 2	6 (15%)	6 (15.38%)	4 (10.25%)	0.555
	Diffuse			6 (15%)		2 (5.12%)	0.189
T Wave Inversion	Specific	16 (40%)	7 ± 3	1 (2.5%)	11 (28.20%)	10 (25.64%)	**0.004**
	Diffuse			15 (37.5%)		1 (2.56%)	**0.001**
Normal Q Wave		10 (25%)	3 ± 2		3 (7.69%)		0.086
Poor R progression	Specific	8 (20%)		5 (12.5%)	6 (15.38%)	6 (15.38%)	0.666
	Diffuse			3 (7.5%)		0 (0%)	0.211
Conduction Abnormalities	AV Block	3 (7.5%)		3 (7.5%)	4 (10.25%)	1 (2.56%)	0.422
	BBB			0 (0%)		3 (7.69%)	0.308
Sinus Tachycardia		6(15%)			1 (2.56%)		0.120

Vital parameters	TCM (*n*_1_ total = 40)	MI (*n*_2_ total = 39)
Pulse rate (bpm)	85 ± 22(63, 107)	79 ± 8 (70, 87)
SBP (mm/Hg)	125 ± 32 (94, 157)	132 ± 30(102, 162)
DBP (mm/Hg)	76 ± 14(63, 90)	85 ± 13(72, 98)

*n*_1_ total = Total number of Takotsubo Cardiomyopathy (TCM) cases

*n*_2_ total = Total number of Myocardial Infarction (MI) cases

*n*_1_ = TCM Cases with a subtype of (*x)* ECG Property

*n*_2_ = MI Cases with a subtype of (*x)* ECG Property

*p* values in bold are significant (< 0.05)

*AV* atrioventricular, *BBB* Bundle Branch Block

## Discussion

### Epidemiology and clinical features

The term “takotsubo” was officially recognized worldwide by Sato et al. in the year 1991 as a pattern of myocardial stunning due to ischemic heart disease [12]. Several cases were also reported from Europe, North America, and Australia in the subsequent years [13]. It was only in 2006, that TCM was finally recognized as a separate clinical entity and a primary acquired form of cardiomyopathy [14]. This led to further inclusion of the diagnosis of TCM by clinicians across the globe. Although a prevalent disease across six continents [15], limited studies on the epidemiology of Takotsubo cardiomyopathy is available in the South-Asian population. The incidence of TCM has increased all over the world mostly attributed to the increased recognition of the syndrome [16]. In our study 40 TCM cases were analyzed from the in-hospital admissions in the cardiology department from 2013 to 2018. The trend of recognition increased in the later years from just 5 cases identified in 2013–14 to 16 cases identified in 2017–18.

A study by Kow et al. involving 98 patients in a hospital in Singapore showed the prevalence of the disease mostly in postmenopausal women with an average age of 69.3 ± 12.6 years [17]. In another study, conducted in India among 43 patients, 77% of the patients were women with an average age of 60.23 ± 12.1 years [18]. Current published literature on TCM patients states 90% of cases occur between 67 and 70 years and 80% of cases are seen after the age of 50. Postmenopausal women have a tenfold greater risk of developing this disease compared to men [19]. In our study, 87.5% of patients were women with 60 ± 11 years as the average mean age, which was in line with the premature postmenopausal age range of Indian women [20]. Although less frequent in men, physical stresses with underlying systemic conditions like stroke, malignancy and infections were primarily responsible for TCM in all the 5 male cases which were included in our study, in line with the results of Kurisu et al. [21]. The high frequency of TCM among postmenopausal women in our study further reiterates the conclusions derived from global studies and literature. The racial predisposition of the disease is unclear with no large-scale studies done in this field. There is only 1 study which states no significant difference in clinical profile within the Asian and other races [22].

The clinical features seen in most of the TCM cases are either primary where patients present with abrupt features to the casualty or secondary where it occurs because of hospital admission or surgical procedures [22]. The primary TCM cases have features of acute chest pain or dyspnea often indistinguishable from features of acute coronary syndrome (ACS) [23]. The secondary TCM have more evidence of features like arrhythmias, hypotension, and acute pulmonary edema [24]. In our study 48% of patients had dyspnea and 28% patients had chest pain which mimicked features of ACS. 45% patients had clinical features other than ACS and they mostly were affected with underlying systemic conditions like heart diseases, pacemaker implantation, pneumonia, sepsis, stroke, and malignancy all secondary in nature. Patients having emotional stress have higher degree of chest pain and dyspnea [19], which was also seen in our study, where 77% of the 18 cases affected by emotional stress had ACS features.

### Risk factors and triggers

The postmenopausal age group in women itself is a major risk factor, a finding that is present in 75% of the patients in our study. The effect of the absence of estrogen on the sympathetic neurohormonal axis as well as the post-menopausal alteration of endothelial function are explanations for this prevalent finding [25]. According to a large collaborative study including 1109 cases of TCM, hypertension was present in 54% of the patients with a range of 27–83% whereas diabetes mellitus was present in 17% of the patients with a range of 4–34% [26]. In our study, the incidences of these comorbidities align with the global ranges with hypertension being present in 40% of the cases and diabetes mellitus being present in 15% of the cases. The cardiovascular risk factors in TCM patients are due to endothelial dysfunction in the pathophysiology, hinted by the absence of obstructive lesions on coronary angiography in the majority of the patients [27]. This is also observed in our study where only 15% of cases had obstructive lesions which were reported as sub-critical on the coronary angiography report.

While there are innumerable triggers that can cause TCM [4], emotional triggers and infections were the most common triggers seen in our study. Interestingly, TCM may also occur without a triggering event and such idiopathic cases are referred to as “stress—cardiomyopathy” [28]. In our study, 45% of the cases were due to emotional triggers and 27.5% of the cases were due to infections, which is a major type of physical trigger. 27.5% of cases did not have a clear-cut identifiable trigger and were identified as stress cardiomyopathy. We also came across some unique triggers such as a complete heart block following pacemaker implantation (7.5%), ruptured coronary aneurysm (2.5%), post-balloon angiography (2.5%), and even bee stings (2.5%). Even though there have been documented cases of TCM in post-operative periods [29], TCM post-bee stings are quite rare [30] and could possibly be due to the sudden catecholamine spike.

### Pathophysiology

The pathophysiology of Takotsubo cardiomyopathy is still poorly understood due to the complex involvement of the brain–heart axis [31]. While various theories have been put forth to understand the events leading to these changes, the catecholamine theory proposed by Witt Stein et al. is the most popular one [19]. Changes to the emotions exert an effect on the autonomic nervous system causing a release of catecholamines and activation of RAAS which in turn causes the release of NT pro-BNP and Troponin T. The significant elevation of Troponin T in our study supports the Witt Stein theory. Some studies have also indicated that local catecholamine excess is responsible for calcium mishandling leading to cardiotoxicity [32].

### Vitals and lab parameters

Pulse rate with SBP and DBP was measured in all 40 cases of TCM and compared with 39 cases of MI. The increased pulse rate among TCM patients compared to MI cases is seen here which is also seen previously in other similar studies [33]. The mean blood pressure values of TCM cases are lower than MI in accordance with a previous study that involved 1750 patients of the International Takotsubo Registry [7]. Troponin T is elevated which is considered a universal biomarker being raised in both TCM and MI cases [6]. NT pro-BNP which is also markedly elevated in the TCM cases is of special significance since the level of rise is 3–fourfold more than in the MI cases [7, 22, 33]. The increased NT pro-BNP is correlated with excessive catecholamine release and LV systolic dysfunction [34].

### ECG characteristics of TCM and MI

A 12-lead ECG with normal standardization and rate was carefully analyzed for all 40 TCM and 39 MI patients as a baseline investigation to detect abnormalities in electrical conduction. The results of both groups were then compared in every parameter.

**Fig. 1:**
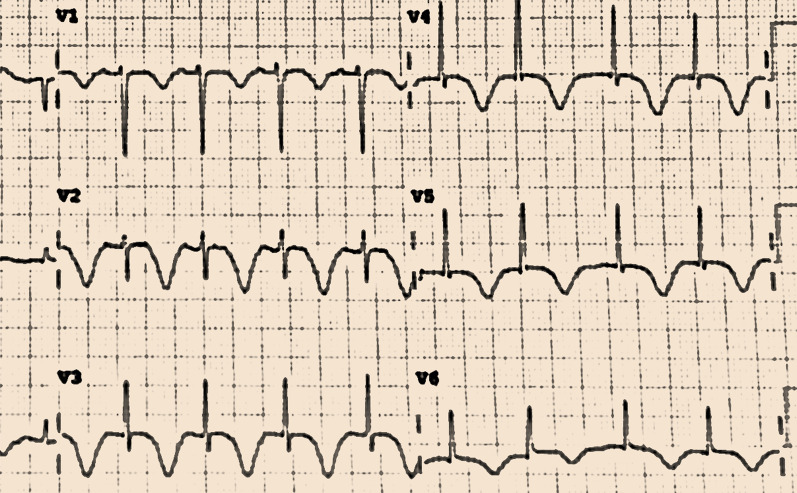
ECG recording in a patient diagnosed with Takotsubo Cardiomyopathy with pre-existing mixed connective tissue disorder showing Q waves with ST Elevation and T Wave Inversions in leads V2-V5

One of the oldest studies done comparing ECG findings of TCM with MI was in 2003 on 13 patients in Japan showed evidence of less frequency of abnormal Q waves, absent reciprocal limb lead elevation, and ST-segment elevation more in the anterolateral leads (V4-V6) than anterior leads (V1–V3) in TCM patients [35] (Fig. 1). In another study involving 15 TCM cases in Japan, the signs of T wave inversion with QT interval prolongation are evident [36]. Distinct patterns were identified in the ECG characteristics when we matched 40 cases of TCM with 39 cases of MI in our study: (1) There is equal frequency of ST segment elevation and T wave inversion in TCM cases with T wave inversion evident in a greater number of leads [37], (2) QTc was prolonged in only 37.5% of TCM cases unlike numerous studies which supported the prolonged QTc to be a more sensitive parameter [35–37], (3) Normal Q waves are present in TCM (25%), whereas MI cases have more frequent abnormal Q waves [35], (4) Reciprocal limb lead ST elevation is present in MI cases more frequently than TCM but they are not absolute in character, (5) ST segment depression is more prevalent in TCM cases than MI cases which deviates from earlier studies [38], (6) T wave inversion in TCM is a more significant with diffuse pattern lead involvement [39], (7) Poor R wave progression is seen in 20% of TCM cases compared to 15% of MI cases showing that TCM can have more evidence of poor R wave progression than MI [40] and (8) Complete heart blocks (CHB) are more evident in TCM [41] compared to bundle branch blocks ( BBB) [42], with none of the cases in our study showing BBB. All these conclusions prove that ECG characteristics may vary in different cases of TCM and MI and they do not form a sacrosanct criterion to come to a diagnosis on a sole basis. Therefore, some studies state that a detailed clinical profile along with coronary angiography is a better way for TCM identification [43].

While we study the literature, we must take into consideration that our study is based in India. The mortality rate due to cardiovascular disease (CVD) is 272 per 100,000 population in India which is higher than the global average of 235 per 100,000 population. Deaths due to CVD occur across all the socioeconomic backgrounds of the nation with the lower socioeconomic group receiving poorer outcomes due to delay in optimal therapy [44]. With TCM being a close mimic of ACS especially among postmenopausal women who form a major section of the population affected by CVD deaths [44] we require an investigation that is economical and available in all the resource settings of our country [45]. Hence in stable patients, we consider that an ECG within 12 h of symptom onset as a baseline investigation for TCM identification is an appropriate investigation in an Indian resource setting as supported by some other studies [46]. We also suggest additionally a detailed clinical profiling of every patient based on routine history, vitals, and basic lab parameters. It can help us narrow down the diagnosis to TCM or ACS which can avert the requirement of an early invasive CAG since it does not impact survival [47]. The use of selective referral strategy of performing computed tomography (CT) CAG in stable patients before invasive CAG is also recommended since it helps increase diagnostic yield and is cost-effective [48]. We have followed the same protocol in all the stable TCM cases analyzed in this study. Seven patients with TCM were seen to have a resolution of these ECG changes at their follow-up visit in our study, hence ECG can also be used for retrospective diagnosis of TCM in patients with a missed diagnosis [49].

### ECHO, follow-up, and CAG

**Fig. 2:**
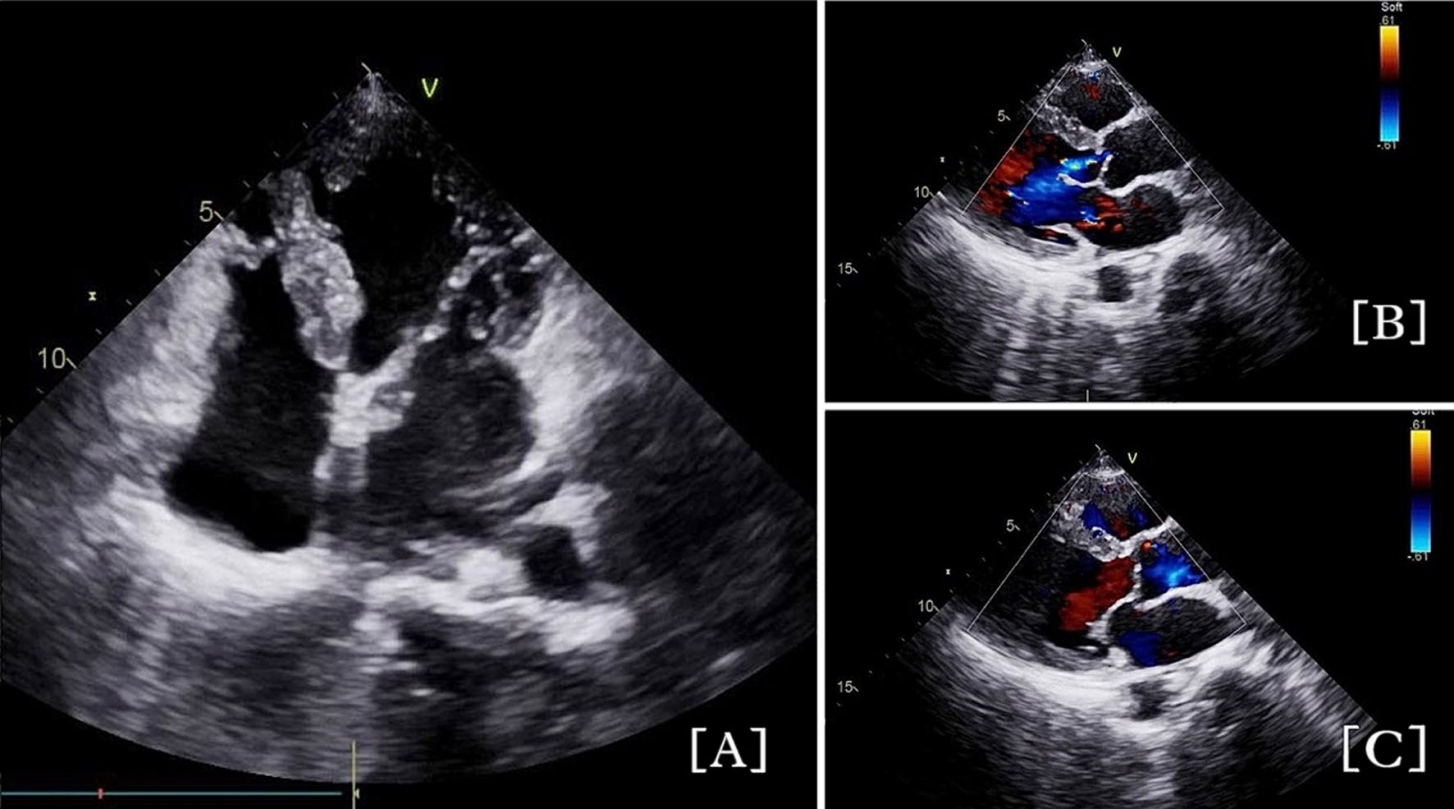
Echocardiogram Images in patients with Takotsubo Cardiomyopathy. **A** ECHO of a patient after a Bee Sting showing Apical Trabeculation, mild Left Ventricular Systolic Dysfunction, and hypokinetic Left Ventricle. **B** Doppler ECHO with Systolic Dysfunction. **C** Doppler ECHO with Normal Diastolic Function

The transthoracic echocardiography though not confirmatory helps to identify the unique changing cardiac function and hemodynamics of TCM [50]. LV myocardial dysfunction seen in 45% of patients in our study is identified by wall motion abnormalities (WMAs). They are seen in mid-ventricular segments compared to apical segments, signifying ventricular dysfunction as the cause of TCM [51] (Fig. 2). The role of sympathetic stimulation in left ventricular dysfunction is controversial [52]. Mitral regurgitation in various grades is seen in 17 out of 18 cases of LV dysfunction in our study and is one of the major complications of TCM. The possible independent mechanisms causing TCM is apical tethering and systolic anterior motion of the mitral valve [53]. Ischemic heart disease evident in 20% of the cases is mostly associated with old age, elevated NT pro-BNP, and low ejection fraction which is also seen in our study with a mean ejection fraction of 43 ± 9% [50]. RV dysfunction is seen only in one case of our study and provides additional findings for the report [54].

The follow-up ECHO shows the resolution of LVEF in 75% of patients seen in various other studies [55]. Additionally, correction in the RWMA in 75% of patients as seen in our study is a common finding in TCM [3]. Hence, ECHO is an ideal non-invasive imaging technique that can be used as a first-line investigation in emergency situations due to its increased availability compared to a CAG setup. Its application has come to the forefront in situations where CAG is contraindicated [50].

The CAG is the most specific investigation for TCM. The absence of coronary artery disease on invasive CAG is a sign of TCM in most cases with exceptions [19]. Although in our study 60% of cases had a normal CAG as expected in a TCM case, 25% of cases had a mild stenosis and 15% had sub-critical stenosis indicating a CAD. This is in line with literature where CAD in TCM is a predictor of 30-day mortality [56].

### Hospitalization, recovery rates, and MACEs

The average duration of stay in the hospital for the patients enrolled in our study was found to be 5 ± 2.96 days due to the extensive investigative work-up and observation. Major studies show that the duration of stay is increased in secondary causes of TCM compared to primary causes [57, 58] which was also a subtle observation in the study. 25% of patients also had a history of previous hospitalizations due to other systemic illnesses. 12.5% of cases had repeat hospitalizations mostly attributed to the recurrence of the disease which was 2.7%. The global reference range for recurrence is 1 to 6% and the rate seen in our study fell under the global average [59]. Compared to ACS cases which require rehospitalization in 1 in 5 cases, TCM cases have a lower repeat hospitalization rate of 1 in 9 cases post 30-day discharge [58]. The secondary causes of TCM were responsible for repeat hospitalization more than the primary causes in our study which matches with other literature [58]. There was an increase in the hospitalization trend which correlates to increased recognition of TCM [16, 60] or identification of TCM as an acute critical illness due to the increased global mortality burden of cardiomyopathy [61].

Though the mortality for TCM is generally low, the 6-month mortality in our study was found to be 7.5%. These patients were found to also have septicemia, septic shock, and aspiration pneumonia along with their TCM making the probable cause of death likely due to the underlying comorbid conditions rather than TCM itself [62]. The deaths in 3 out of the 4 cases in our study were due to physical triggers and all of them had a non-cardiovascular origin. Both these trends of mortality are seen in the literature [63]. MACE is defined as a composite of adverse events which predominantly considers all-cause death, nonfatal myocardial infarction, stroke, revascularization procedures, and hospitalization because of heart failure [11]. In this study, we use the MACE components to analyze the complications in TCM patients. The MACEs act as a primary endpoint that helps in risk–benefit ratio calculation and predicting patient outcomes [64].

## Conclusions

Takotsubo cardiomyopathy (TCM) is an acquired clinical entity characterized by transient LV dysfunction. The recent increase in TCM admissions is due to increased global recognition of the disease. Clinical profile analysis of 40 Indian patients with TCM revealed that postmenopausal women (within the age range less than the global mean) and patients with hypertension were commonly affected. The causes of secondary TCM had a similar clinical frequency as primary TCM. Emotional triggers were more responsible than physical triggers, however, the physical triggers led to increased predictive mortality rates. Universal findings of increased pulse rate, elevated Troponin T, and NT pro-BNP were evident. ECG features of diffuse T wave inversions, reversible abnormal Q in addition to ST depression as well as poor R wave progression were seen in TCM in contrast to the specific changes in the leads seen in MI. ECHO showed increased LV dysfunction with MR as a major complication. Follow-up ECHO showed resolution in LVEF and RWMA in most cases. CAG report was normal in 60% of cases but 40% had evidence of mild or subcritical stenosis. The MACE and recurrence rates were also within the standard ranges of global literature. Overall, the study shows that, apart from certain deviations, TCM in India follows trends like the regions of the USA, EU, UK, and East Asia. Despite high reversibility rates in the majority, TCM with complications has a poorer prognosis than MI, and a presentation almost indistinguishable from ACS, proving to be a diagnostic dilemma. Accordingly, the drastic rise in Indian incidence and death rates of ACS-like diseases, along with the lack of substantial literature, necessitates the requirement of TCM studies in our country highlighting the specific clinical profile, diagnosis, and management.

## Limitations of the study

The study's primary limitation was that it was a retrospective, single-center study with a very small sample size. The subjects of TCM could also not be directly compared with the MI subjects since it was nonrandomized. Hence, a comparability of the management of TCM and MI could not be established, which remains to be the future scope of this study. Additionally, data for more than 6-month follow-up was not available. More studies with larger sample sizes are required within the diverse Indian or Southeast Asian population to verify the conclusion obtained from this study.

### Study design

This single-center, retrospective observational study included 40 patients diagnosed with TCM and 39 patients diagnosed with MI, in a tertiary care hospital in South India. The study was approved by the Institutional Ethics Committee (IEC) of Kasturba Medical College and Hospital (IEC Number: 205/2019). Clinical profiles, laboratory parameters, echocardiographic (ECHO), and coronary angiography (CAG) findings were analyzed for patients with TCM. A comparison of electrocardiographic (ECG) data of TCM patients was done with MI patients.

## Data Availability

The data underlying this article will be shared upon reasonable request to the corresponding author.

## Abbreviations

TCM: Takotsubo Cardiomyopathy
ECG: Electrocardiography
ECHO: Echocardiography
MI: Myocardial Infarction
CAG: Coronary Artery Angiography
MACE: Major Adverse Cardiovascular Events
STEMI: ST Elevation Myocardial Infarction
NSTEMI: Non ST Elevation Myocardial Infarction
BBB: Bundle Branch Block
LVEF: Left Ventricular Ejection Fraction
RWMA: Regional Wall Abnormalities
ACS: Acute Coronary Syndrome
LV: Left Ventricle
SBP: Systolic Blood Pressure
DBP: Diastolic Blood Pressure
CVD: Cardiovascular Disease
WMA: Wall motion abnormality
CT: Computed Tomography
